# Research hotspots, context, and future directions of ulcerative colitis comorbid with depression from 2006 to 2025: a bibliometric and visualization analysis

**DOI:** 10.3389/fmed.2026.1868648

**Published:** 2026-08-05

**Authors:** Yang Yang, Yifei Wang, Shuxin Zhang, Guangyao Chen, Shuangshuang Ji, Xincheng Wang, Sheng Li

**Affiliations:** 1 Dongzhimen Hospital, Beijing University of Chinese Medicine, Beijing, China; 2 Department of TCM Rheumatology, China-Japan Friendship Hospital, Beijing, China; 3 Beijing Key Lab for Immune-Mediated Inflammatory Diseases, China-Japan Friendship Hospital, Beijing, China; 4 Xiyuan Hospital, China Academy of Chinese Medical Sciences, Beijing, China; 5 Department of Stomatology, Weifang Nursing Vocational College, Weifang, Shandong, China

**Keywords:** bibliometric analysis, comorbidity, depression, research trends, ulcerative colitis

## Abstract

**Background:**

Depression is highly prevalent in patients with ulcerative colitis (UC), affecting prognosis and patients’ quality of life. While related studies grow rapidly, a systematic review of the development trends in this field is still lacking. Based on this, the present study used bibliometric and visualization analysis methods to systematically examine the research landscape, hot topics, and future directions in the global field of UC comorbid with depression.

**Methods:**

Relevant literature published between January 1, 2006 and December 31, 2025 in the Web of Science Core Collection (WOSCC) and Scopus databases was retrieved. CiteSpace and VOSviewer software were used to analyze countries, journals, authors, references, and keywords, and co-occurrence network maps were generated. For complementary analysis, clinical trial articles published in PubMed over the same time period were retrieved.

**Results:**

A total of 2,069 papers were included in the visualization analysis, with an additional 28 records identified through supplementary searching. In 2021, the number of publications increased significantly and has since remained at a relatively high level. International academic exchanges in UC comorbid with depression research are active, with the United States, the United Kingdom, and Canada being the core research forces in this field. *Inflammatory Bowel Diseases* is the most published and most representative professional journal. Bernstein, Charles N. and Mikocka-Walus, Antonina have published the highest number of studies, while Ananthakrishnan, Ashwin N. and Ford, Alexander C. have the highest academic influence. The co-citation network of references suggests that research in this field is based on backgrounds such as pain management, perceived stress, and psychiatry. Keyword analysis shows that core research hotspots focus on epidemiological studies, drug safety, and side effect management. Multidisciplinary combined treatment involving gastroenterology, psychiatry, and endocrinology is a frontier research direction.

**Conclusion:**

External environmental stressors and intrinsic metabolic dysregulation are jointly reshaping the frontier landscape of this field. Beyond traditional epidemiological investigations, patient quality of life and drug safety have emerged as current research priorities. Future research should focus on generating a greater volume of clinical evidence and promoting multidisciplinary collaborative care, with the goal of improving patients’ quality of life and long-term prognosis.

## 1 Introduction

Ulcerative colitis (UC) is a recurrent, chronic, non-specific inflammatory bowel disease (IBD) characterized by continuous colonic mucosal inflammation, abdominal pain, recurrent diarrhea, and bloody mucoid stools (1). UC not only significantly reduces patients’ quality of life but can also induce serious complications such as intestinal obstruction, perforation, and colorectal cancer (CRC) (2, 3). Currently, the prevalence of UC is on the rise, with approximately 5 million UC patients worldwide in 2023 (4, 5). The large patient population and the recurrent, prolonged disease course will inevitably lead to long-term continuous consumption of medical resources (6). However, UC is not just an organic disease confined to the intestine. Accumulating evidence suggests that patients with UC frequently experience significant psychiatric comorbidities, among which depression is one of the most prevalent and burdensome (7, 8).

Epidemiological data from recent years have indicated that the prevalence of depression among patients with UC ranges from 25 to 60% (9), markedly exceeding that observed in healthy populations and individuals with other chronic diseases. Depression not only exacerbates intestinal inflammation and undermines treatment adherence in UC patients (10, 11), but also elevates the risks of disease recurrence and hospitalization (12), making it a key factor that affecting clinical efficacy and long-term prognosis. UC and depression share complex pathophysiological mechanisms such as hypothalamic–pituitary–adrenal (HPA) axis, immune-mediated inflammation, gut microbiota alterations, and neuroendocrine disturbances (13–15). However, due to marked heterogeneity in patient cohorts, treatment regimens, and assessment instruments, a unified theoretical framework and standardized diagnostic and treatment pathway have not yet been formed in this field.

With the continuous accumulation of basic and clinical research, UC comorbid with depression has received widespread global attention. Bibliometric analysis and visualization analysis rely on scientometric tools to systematically sort out the disciplinary development context, identify core research forces, and track hotspot evolution and frontier directions (16–18). Currently, panoramic, long-term, multi-dimensional visualization studies in the field of UC comorbid with depression are still relatively lacking. Based on this, the present study used the Web of Science Core Collection (WOSCC) and Scopus as the primary databases for analysis and cross-validation, supplemented by additional searches in PubMed. Through bibliometric and visualization analyses of the literature published from 2006 to 2025, we delineated the knowledge structure and research landscape of the field, and identified current research hotspots and core themes. This study is expected to provide theoretical references for the integrated diagnosis and treatment of comorbid conditions, as well as for future research directions.

## 2 Materials and methods

### 2.1 Data sources and search strategies

The search query for WOSCC was: TS = (“ulcerative colitis” AND (depression OR “depressive disorder” OR “depressive symptom*”)). The time span was set from January 1, 2006 to December 31, 2025, and the document types were limited to Article and Review Article. Prior to 2006, research on this comorbidity was relatively scarce, therefore, following the common practice in bibliometric studies, we selected 2006 as the starting point. After applying the inclusion and exclusion criteria, a total of 1,028 articles were obtained. They were exported in Plain Text File and Tab-delimited File formats for analysis using CiteSpace (version 6.4. R1; Drexel University, Chaomei Chen) and VOSviewer (version 1.6.20; Leiden University,van Eck and Waltman). The search query for the Scopus was: TITLE-ABS-KEY(“ulcerative colitis” AND (depression OR “depressive disorder” OR “depressive symptom*”)), with the same time,document type restrictions and screening criteria, yielding 1,595 articles, which were exported as CSV files.

Excel was used to deduplicate the literature from the two databases based on the DOI field. For records lacking a DOI field, we first identified potential duplicates by matching titles and then confirmed them by comparing author names. For records with highly similar titles but discrepancies in author information, we further consulted the original abstracts to verify whether they referred to the same publication. The deduplication process was performed independently by two researchers, after which their results were cross-validated. Any disagreements were resolved through discussion with a third researcher. After removing 554 duplicate articles, a final dataset of 2,069 articles was obtained for bibliometric and visualization analysis.

**Figure 1:**
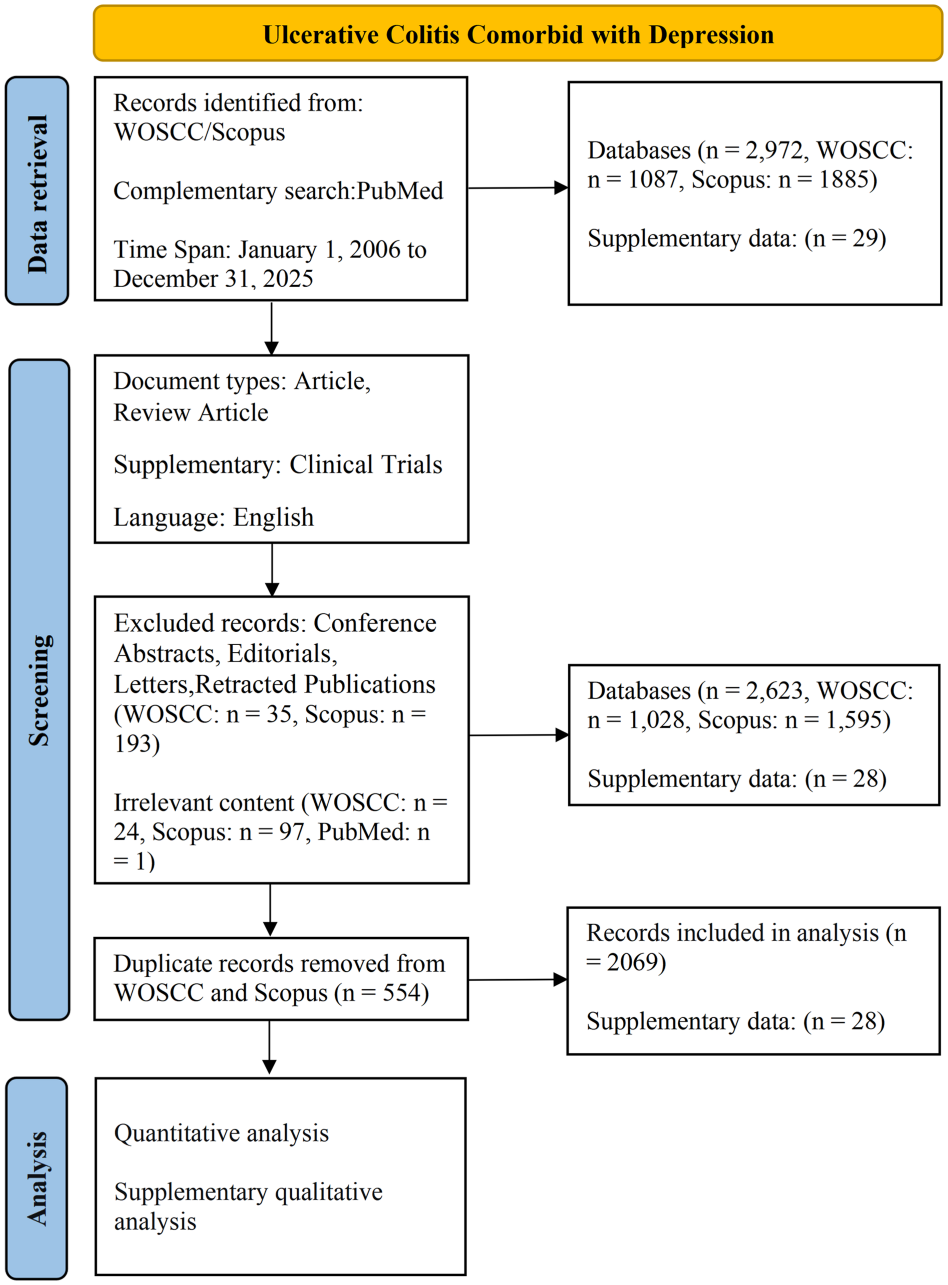
Literature retrieval flow chart.

Meanwhile, PubMed was incorporated as a supplementary database to further explore clinical research trends. The search query for PubMed was: (“ulcerative colitis”[tiab] AND (depression[tiab] OR “depressive disorder”[tiab] OR “depressive symptom*”[tiab])), with the same time span and document type restricted to Clinical Trial. A total of 28 records were included in the qualitative analysis. The literature search and collection process is illustrated in Figure 1, and the detailed search strategies are provided in Supplementary Table S1.

### 2.2 Data analysis

The bibliometric and visualization analyses in this study were completed based on standard analysis software. First, WPS Excel 2023 was used to collate and analyze the basic characteristics of the included literature, including annual publication trends, distribution of high-frequency keywords, and rankings of journal and author publication volumes. Then, CiteSpace (version 6.4. R1; Drexel University, Chaomei Chen) software was employed for further bibliometric analysis and visualization network construction (17). The time span was set from 2006 to 2025, with a slice length of 1 year. Node types were sequentially selected as Keyword, Institution, Country, Cited Journal, Cited Author, and Cited Reference. For node selection, the *g*-index (k = 10) and TopN (*N* = 50) criteria were applied to each time slice. Network pruning was performed using the Pathfinder algorithm, combined with both “Pruning sliced networks” and “Pruning the merged networks” to optimize the global network structure. Following the keyword co-occurrence analysis, burst detection was conducted with a Gamma (*γ*) value of 1 and a minimum duration of 2 years. Cluster analysis was performed using the Log-Likelihood Ratio (LLR) algorithm.

To more accurately delineate the intrinsic relationships among countries, authors, and keywords, we performed a complementary analysis using VOSviewer (version 1.6.20; Leiden University, developed by N. J. van Eck and L. Waltman). Cooperative networks were constructed separately for authors, institutions, and countries, while a co-occurrence network was constructed for keywords. Network construction employed the Full Counting method, and normalization was performed using the Association Strength algorithm. For clustering, the Resolution parameter was set to 1.0, and the Minimum cluster size was set to 5. In the resulting cluster maps, node size represents its weight (e.g., publication count or co-occurrence frequency), and node color indicates its cluster assignment (16). In addition this study used Scimago Graphicato (version 1.0.38; SCImago Research Group, Hassan-Montero et al) to geographically visualize the country collaboration network, thereby intuitively illustrating the geographic distribution of research and international collaboration trends (19).

## 3 Results

### 3.1 Publication volume trend

**Figure 2:**
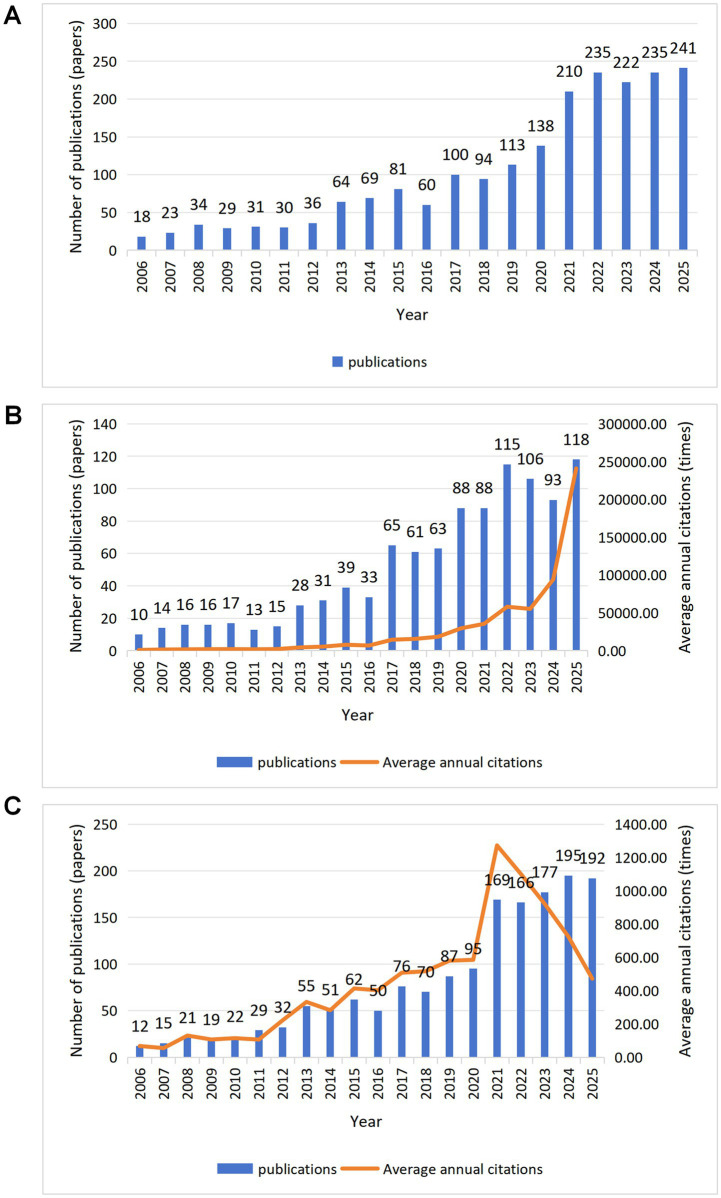
Analysis of annual publication volume and average annual citations from 2006 to 2025. **(A)** Bar chart showing annual publication counts after duplicate removal from two combined databases between 2006 and 2025. The horizontal axis represents publication year, and the vertical axis denotes the annual number of included publications. **(B)** Dual-axis chart illustrating annual publication volume and average annual citations for literature retrieved from the WoSCC. Blue bars represent annual publication quantity, and the orange line indicates average annual citations. **(C)** Dual-axis chart illustrating annual publication volume and average annual citations for literature retrieved from Scopus. Blue bars represent annual publication quantity, and the orange line indicates average annual citations. Average annual citations: Total citations of publications published in a given year divided by the number of natural years elapsed since publication.

The annual publication volume for research on UC and depression is presented in Figure 2A. From 2006 to 2012, the field remained in its nascent stage, with a low annual output averaging approximately 29 articles. The year 2013 marked a pivotal turning point, as publication numbers surged from 36 in 2012 to 64, a nearly 80% increase signaling growing scholarly attention to this topic. A second sharp rise occurred in 2021, and output has since remained at a relatively high level overall, with an average of 228.06 articles per year over the past five years, showing strong research momentum. The annual publication volumes retrieved separately from WoSCC (Figure 2B; Supplementary Table S2) and Scopus (Figure 2C; Supplementary Table S3) exhibited overall upward trajectories consistent with the combined dataset. To address the inherent limitation that older publications tend to accumulate more total citations over time, we employed average annual citations as a normalized metric to objectively evaluate yearly academic impact. For WOSCC, average annual citation values remained low and stable before 2020, then rose rapidly thereafter. The average annual citations for Scopus papers peaked around 2021 and gradually declined in subsequent years. This decline may be attributed to insufficient time for citation accumulation among recently published works, or to differences in journal coverage and publication scope between the Scopus database and WOSCC. These upward trends in both publication volume and average citation impact collectively indicate that research on UC with comorbid depression is gaining increasing scholarly recognition and is becoming an active and impactful area of investigation.

### 3.2 Country output and cooperation

**Figure 3:**
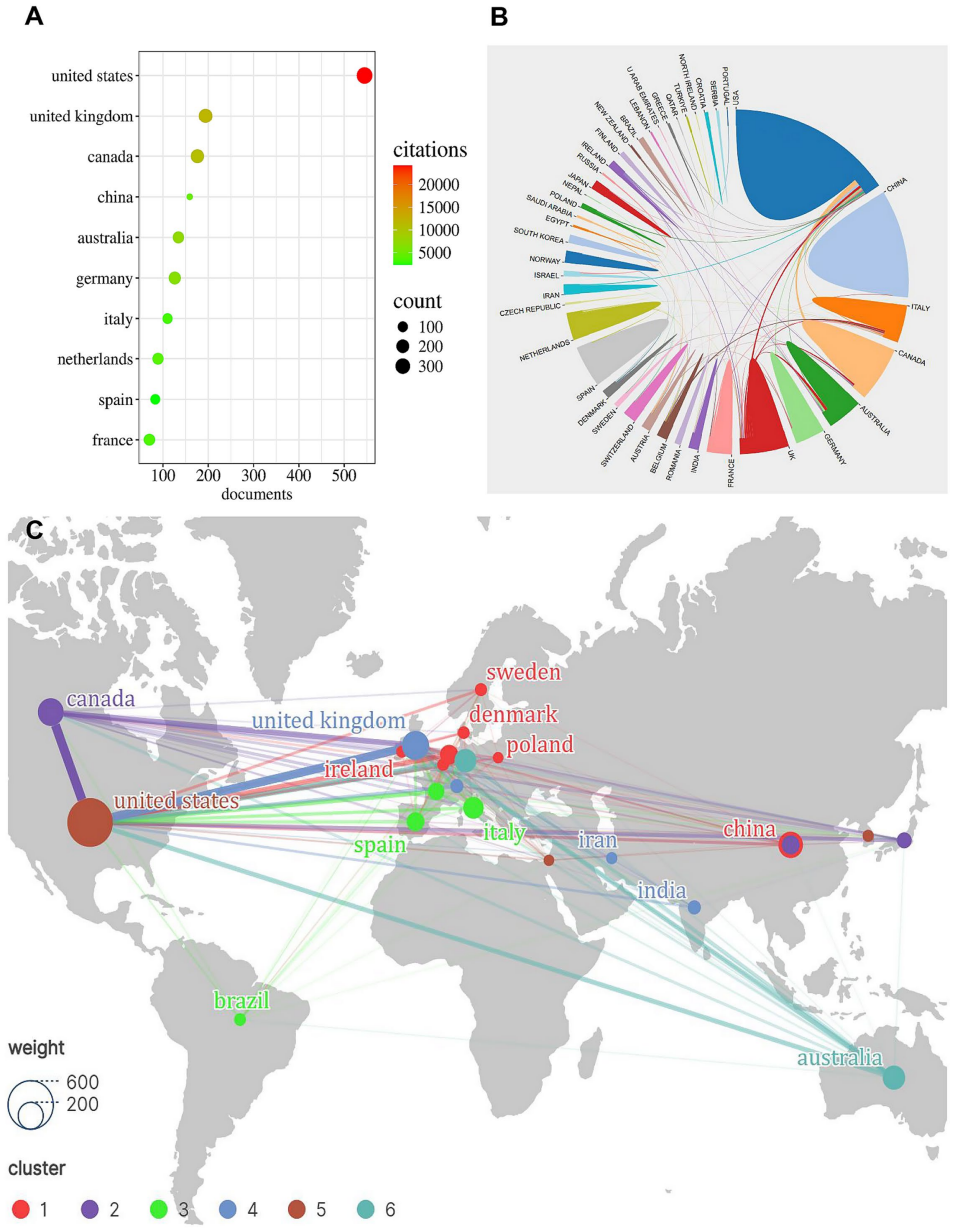
Country output and cooperation. **(A)** Bubble chart of publication volume and citation intensity by country. The horizontal axis represents the publication volume of each country, bubble size represents publication scale, and the color gradient (from green to red) represents citation intensity, with darker red indicating higher citation intensity. **(B)** Chord diagram of country cooperation network. Each arc represents a country, and arc length is proportional to the country’s total number of collaborations. Chords indicate collaborative relationships between two countries, and the width of chords is proportional to the collaboration strength. **(C)** Global cooperation network map. Node size represents the publication volume of each country, node color represents different cooperation clusters (1–6), and line thickness represents the intensity of cooperation between countries.

Using country as the analytical dimension, we performed statistical and visual analyses of publication characteristics for research on UC and depression, the results are summarized in Table 1 and Figure 3. The United States ranked first globally in both the number of publications (545) and citation frequency (24,155 times), making it the core research force in this field (Figure 3A). The United Kingdom and Canada followed closely, also ranking among the top countries on both metrics. The chord diagram (Figure 3B) further indicated that global research has formed a multinational cooperation network with the United States, the United Kingdom and Australia serving as central hubs. Active international academic exchanges suggest that this cooperative network has become highly mature. The world map overlay of cooperation networks (Figure 3C) identified six distinct clusters among global research. Among them, the United States, the United Kingdom, and Canada—representing major European and American countries—served as central nodes, linking closely collaborating clusters across Europe and North America. Meanwhile, China, India, Australia, and other Asia-Pacific and emerging market countries have emerged as key nodes connecting Eastern and Western research, enabling the collaborative network to extend across all major research regions worldwide.

**Table 1:** Top 10 countries by publication volume.

Rank	Country	Documents	Citations	Total link strength
1	United States	545	24,155	360
2	United Kingdom	194	11,753	239
3	Canada	176	10,797	214
4	China	159	4,851	44
5	Australia	134	7,384	135
6	Germany	126	6,527	166
7	Italy	110	3,310	95
8	Netherlands	89	4,006	129
9	Spain	83	2,323	91
10	France	70	3,621	143

### 3.3 Journal publication volume and impact

**Figure 4:**
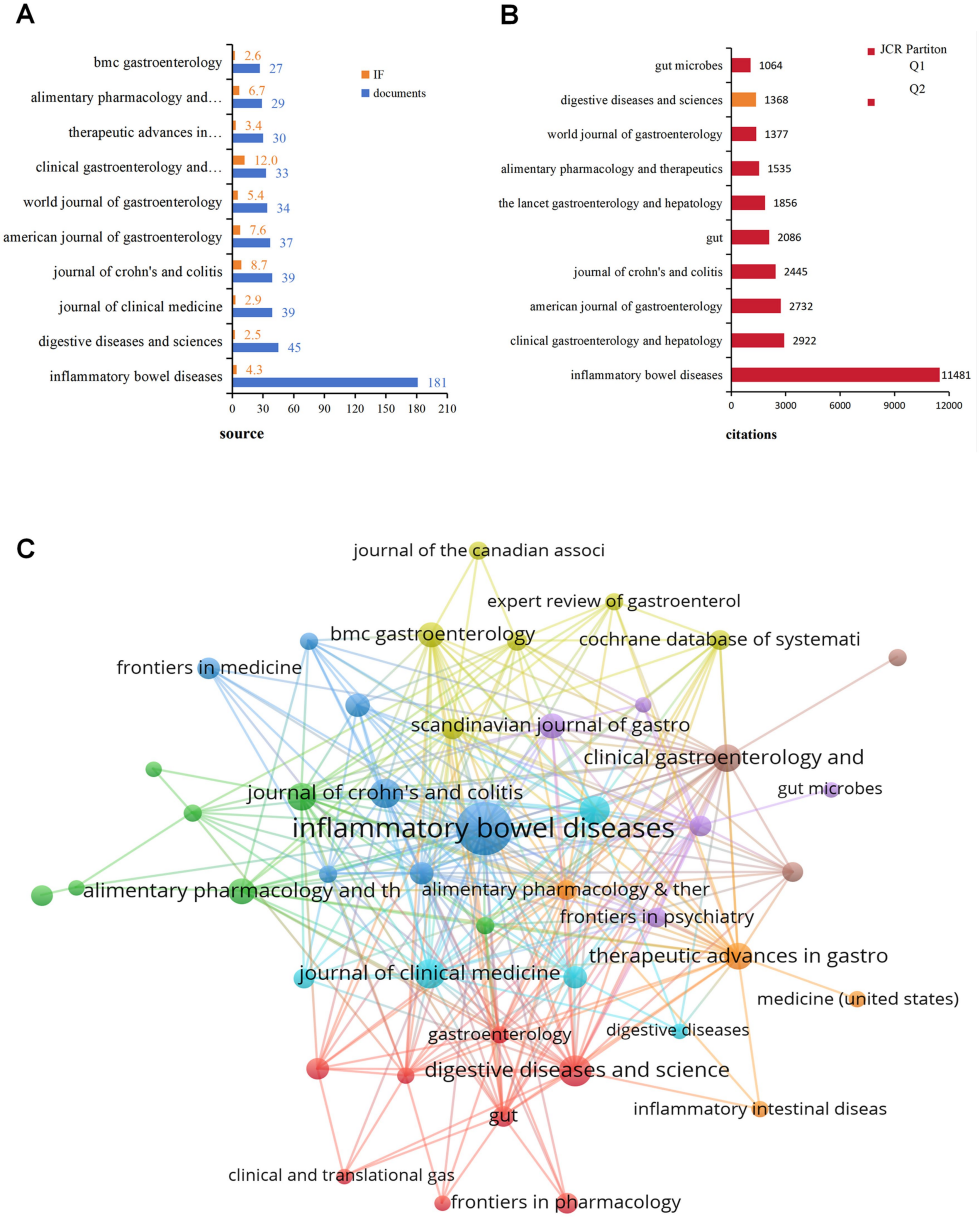
Journal publication volume and impact. **(A)** Clustered bar chart of the top 10 journals by publication volume and their impact factors. The horizontal axis represents the specific values of publication volume and impact factor, the vertical axis labels the names of the top 10 journals by publication volume, orange bars represent impact factors, and blue bars represent the number of published articles. **(B)** Clustered bar chart of the top 10 journals by citation frequency and JCR quartile. The horizontal axis represents total citation frequency, bar color represents the journal’s JCR quartile (red for Q1, orange for Q2). **(C)** Journal cooperation network map. Node size represents the journal’s publication volume, node color represents different cooperation clusters, and connecting lines represent cooperative relationships between journals.

Statistical analysis of journals publishing the articles was performed, and the results are shown in Figure 4. *Inflammatory Bowel Diseases* published 181 articles, making it the most prolific and leading specialty journal in this field. *Clinical Gastroenterology and Hepatology* had a limited number of publications but has a high impact factor (IF) of 12.0, serving as a key outlet for influential research findings (Figure 4A). Journal citation analysis showed that *Inflammatory Bowel Diseases* is the core journal with the highest total citation frequency (11,481 times). Top gastroenterology journals, including *Clinical Gastroenterology and Hepatology* (2,922 times) and *American Journal of Gastroenterology* (2,732 times), are highly cited and ranked in the Journal Citation Reports (JCR) Q1 quartile, underscoring their role as outlets for high-impact research. In terms of citation rate, *The Lancet Gastroenterology* and *Hepatology* had the highest average citation rate per article, followed by *Gut Microbes* and *Gut*, indicating that the research results published in them have greater influence (Table 2). The journal co-citation analysis (Figure 4C) network formed multiple stable clusters: an IBD journal cluster centered on *Inflammatory Bowel Diseases* and *Journal of Crohn’s and Colitis*, a top gastroenterology journal cluster centered on *Gut* and *American Journal of Gastroenterology*, and an interdisciplinary journal cluster represented by *Frontiers in Medicine, Frontiers in Psychiatry* and *Journal of Psychosomatic Research.*

**Table 2:** Top 10 journals by average citation frequency.

Rank	Journals	Documents	Citations	Average citation frequency
1	The lancet gastroenterology and hepatology	9	1856	206
2	Gut microbes	8	1,064	133
3	Gut	16	2086	130
4	Journal of the American academy of dermatology	8	951	119
5	Gastroenterology	10	1,009	101
6	British journal of dermatology	11	975	89
7	Clinical gastroenterology and hepatology	33	2,922	89
8	American journal of gastroenterology	37	2,732	74
9	Inflammatory bowel diseases	181	11,481	63
10	Journal of crohn’s and colitis	39	2,445	63

### 3.4 Analysis of author publication volume and impact

**Figure 5:**
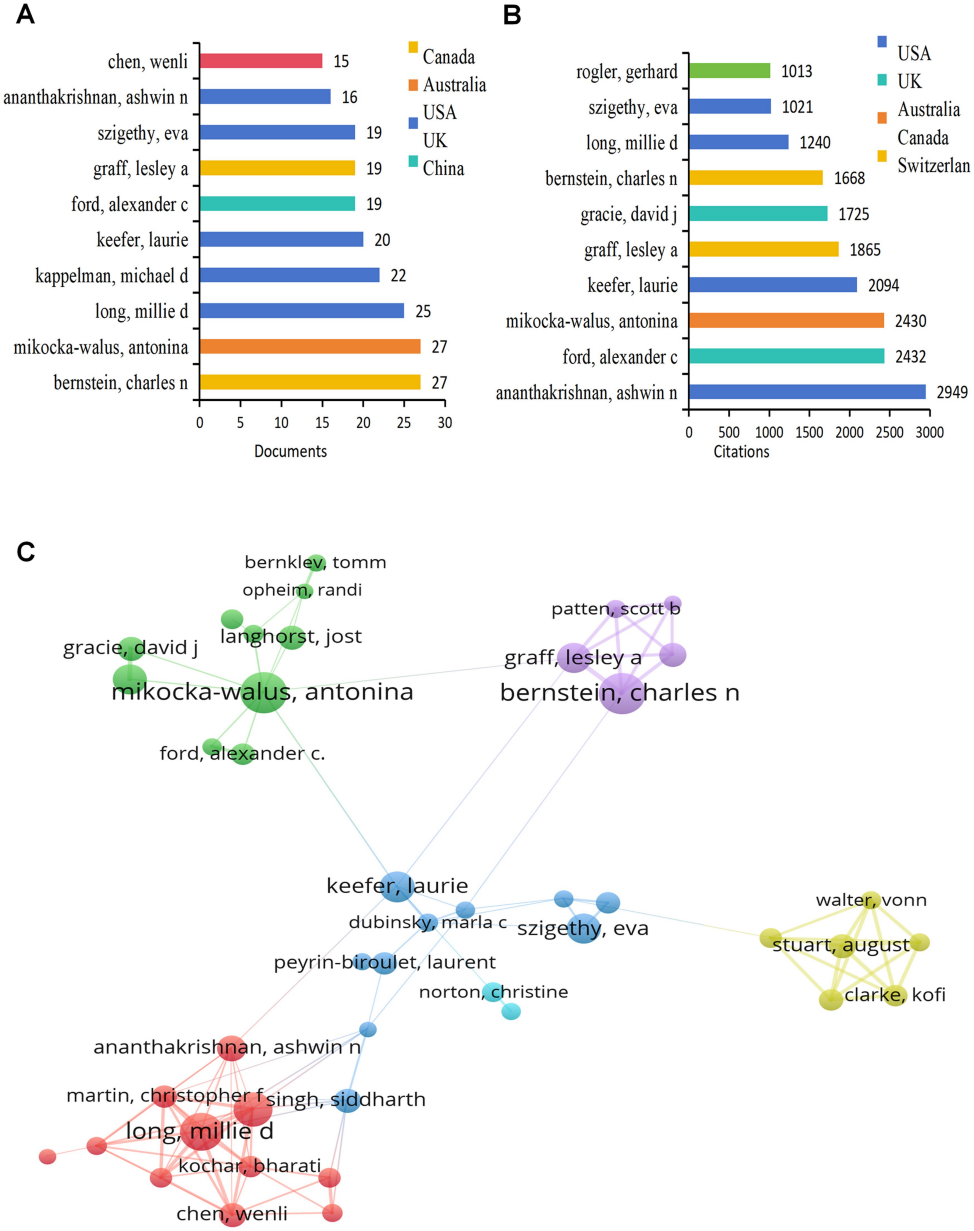
Analysis of author publication volume and impact. **(A)** Bar chart of the top 10 authors by publication volume. The horizontal axis represents the number of publications, and the color represents the author’s country/region. **(B)** Bar chart of the top 10 authors by citation frequency. The horizontal axis represents the total citation frequency. **(C)** Author co-occurrence network. Node size represents the author’s publication volume, node color represents different cooperation clusters, and connecting lines represent cooperative relationships between authors.

Analysis of publishing authors is shown in Figure 5A. Bernstein, Charles N. (27 articles), Mikocka-Walus, Antonina (27 articles), Long, Millie D. (25 articles), and Kappelman, Michael D. (22 articles) have published the highest number of articles and are core researchers in UC comorbid with depression. As shown in Figure 5B, authors’ publication volume does not completely overlap with their citation frequency. Authors such as Ananthakrishnan, Ashwin N. (2,949 times), Ford, Alexander C. (2,432 times), and Mikocka-Walus, Antonina (2,430 times) have higher citation frequencies, indicating that they have stronger academic influence. The author cooperation co-occurrence network (Figure 5C) formed six cooperation clusters. These include a green cluster centered on Mikocka-Walus, Antonina and Gracie, David J.; a purple cluster centered on Bernstein, Charles N. and Graff, Lesley A.; a blue cluster centered on Keefer, Laurie and Szigethy, Eva; and a red cluster centered on Long, Millie D. and Chen, Wenli. Each cluster has close internal cooperation, and there is a small amount of cross-cluster cooperation between clusters.

### 3.5 Analysis of reference contribution

**Figure 6:**
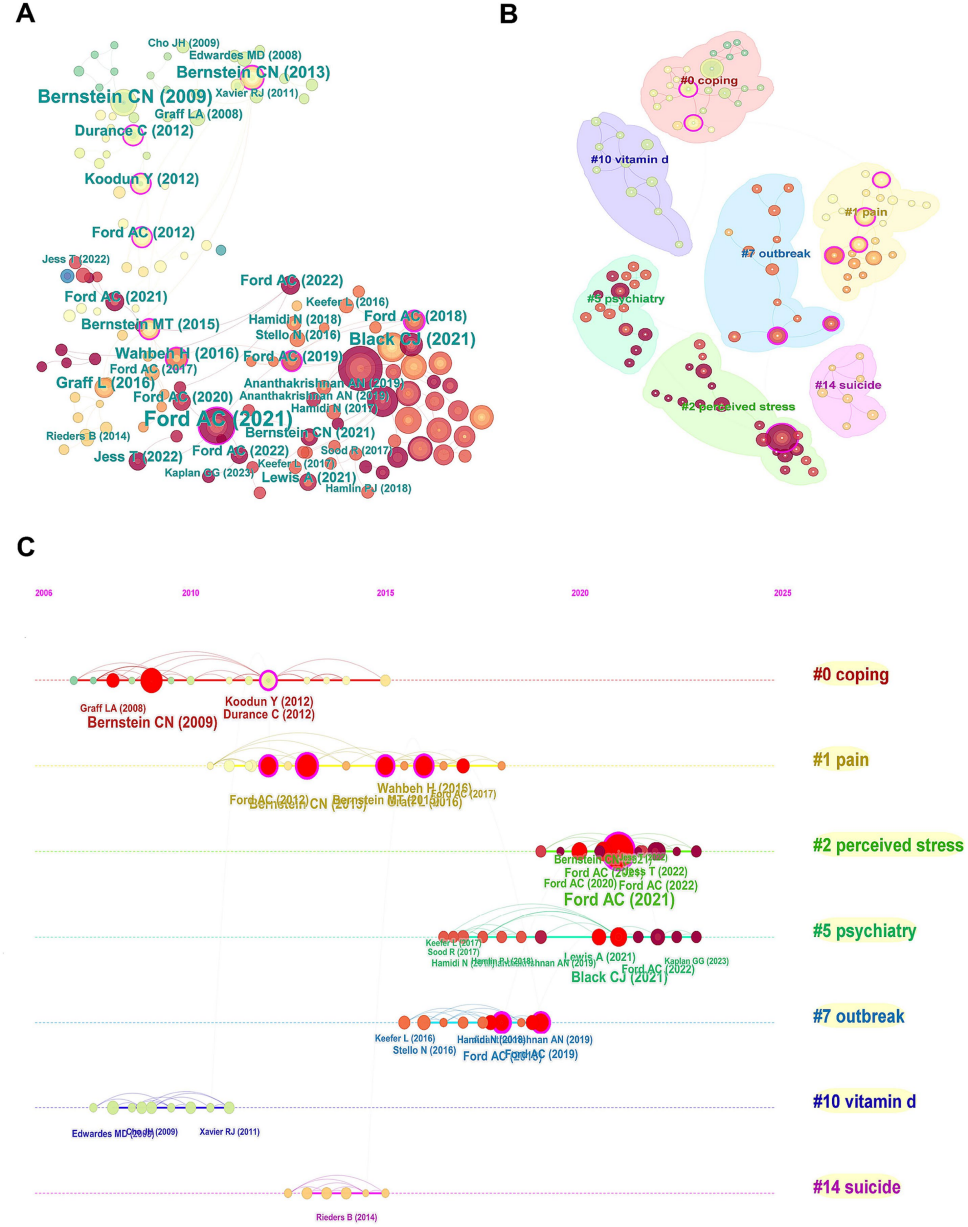
Analysis of reference contribution. **(A)** Reference co-citation network map. Node size represents the citation frequency of the literature, node color corresponds to the publication year, and connecting lines represent co-citation relationships. **(B)** Reference co-citation clustering map. Different colors represent different research theme clusters. **(C)** Reference co-citation timeline diagram. The horizontal axis represents the publication year, the vertical axis represents research theme clusters, and node size represents citation frequency.

The literature co-citation network analysis (Figure 6) showed that references have formed a highly interconnected knowledge network (Figure 6A), with core highly cited publications constituting the knowledge base of the field. Notable systematic reviews, including Barberio B (2021) (20), Bisgaard TH (2022) (21), and Mikocka-Walus A (2016) (22), have addressed the prevalence of IBD, the status of anxiety and depression in IBD populations, and shared pathogenic mechanisms. These publications form core nodes within the co-citation network. The top 10 most cited articles are listed in Table 3. The co-citation network clustering analysis (Figure 6B) revealed six major clusters, indicating the core backgrounds and theoretical cornerstones of research on UC with comorbid depression: #0 coping, #1 pain, #2 perceived stress, #5 psychiatry, #7 outbreak, #10 vitamin D, #14 suicide. The timeline visualization (Figure 6B) further presented the evolutionary trajectory of each cited theme. In early research (2008–2015), coping style and pain management were the predominant cited themes. During the mid-term, perceived stress and psychiatry emerged as the main research backgrounds, indicating a growing scholarly focus on mental and psychological states. Since 2020, outbreak-related stress and suicide risk have become new research backgrounds.

**Table 3:** Top 10 most cited documents.

Rank	Literature	Count
1	Barberio B, 2021, LANCET GASTROENTEROL, V6, P359, DOI: 10.1016/S2468-1253(21)00014-5	58
2	Bisgaard TH, 2022, NAT REV GASTRO HEPAT, V19, P717, DOI: 10.1038/s41575-022-00634-6	29
3	Mikocka-Walus A, 2016, INFLAMM BOWEL DIS, V22, P752, DOI: 10.1097/MIB.0000000000000620	28
4	Neuendorf R, 2016, J PSYCHOSOM RES, V87, P70, DOI: 10.1016/j.jpsychores.2016.06.001	23
5	Ng SC, 2017, LANCET, V390, P2769, DOI: 10.1016/S0140-6736(17)32448-0	23
6	Bernstein CN, 2019, INFLAMM BOWEL DIS, V25, P360, DOI: 10.1093/ibd/izy235	21
7	Alatab S, 2020, LANCET GASTROENTEROL, V5, P17, DOI: 10.1016/S2468-1253(19)30333-4	20
8	Gracie DJ, 2018, GASTROENTEROLOGY, V154, P1635, DOI: 10.1053/j.gastro.2018.01.027	19
9	Gracie DJ, 2017, LANCET GASTROENTEROL, V2, P189, DOI: 10.1016/S2468-1253(16)30206-0	19
10	Mikocka-Walus A, 2016, CLIN GASTROENTEROL H, V14, P829, DOI: 10.1016/j.cgh.2015.12.045	17

### 3.6 Analysis of co-cited reference authors

**Figure 7:**
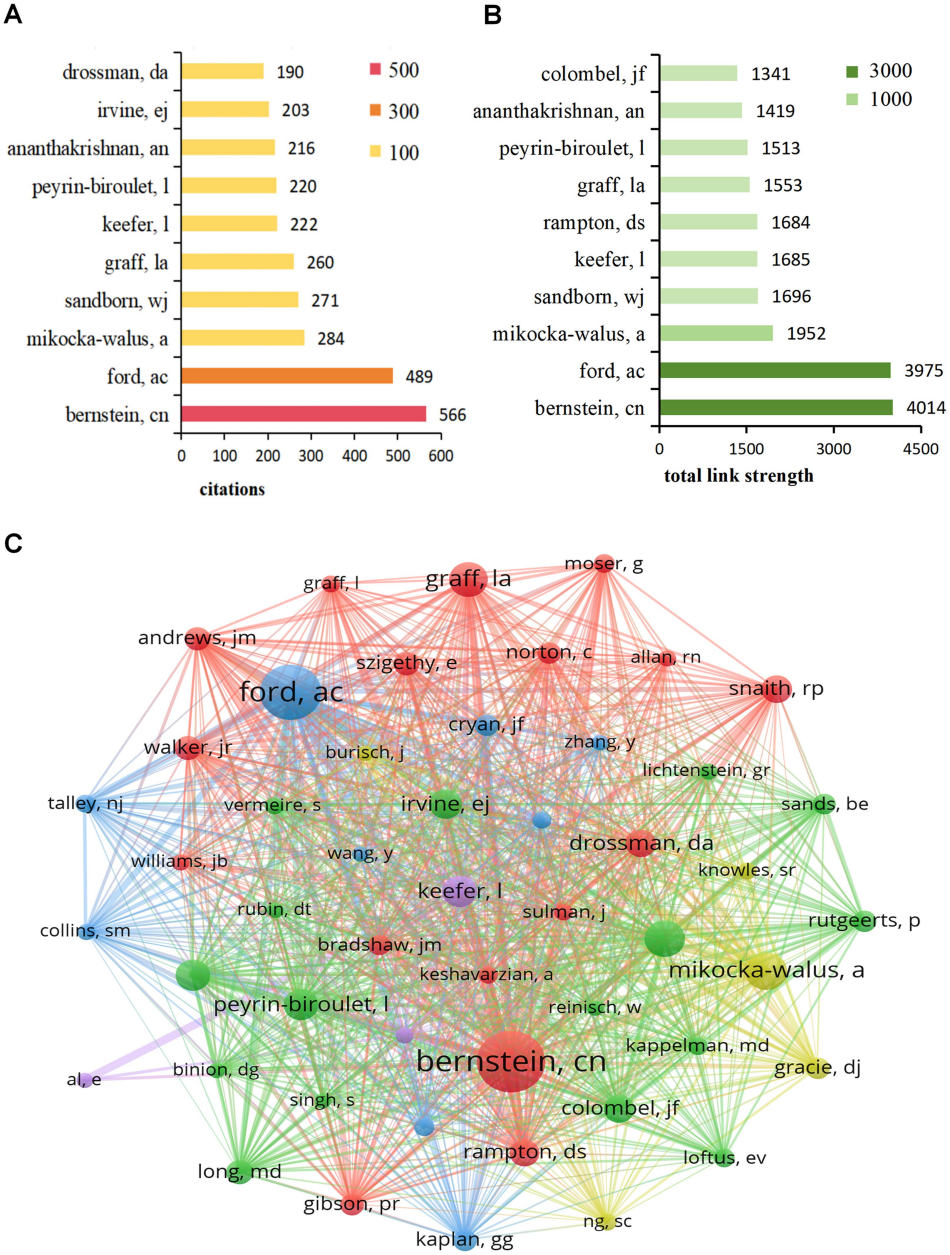
Analysis of co-cited reference authors. **(A)** Bar chart of the top 10 reference authors by total citation frequency. Bar color corresponds to the citation frequency range, and bar length represents the author’s total citation frequency. **(B)** Bar chart of the top 10 reference authors by total link strength. Bar color corresponds to the link strength range, and bar length represents the author’s total link strength in the co-citation network. **(C)** Reference author co-citation network map. Node size represents the author’s citation frequency, node color represents different clusters, connecting lines represent co-citation relationships between authors, and line thickness represents co-citation intensity.

Author co-citation analysis is an effective method for revealing the background origins and knowledge structure of a research field. As shown in the citation frequency analysis in Figure 7A, Bernstein CN (566 times) and Ford AC (489 times) rank first and second in citation frequency among reference authors, with a substantial lead over others, distinguishing them as the core founders of the knowledge system in this field. Mikocka-Walus A (284 times), Sandborn WJ (271 times), and Graff LA (260 times) constitute the core team of highly cited authors. Figure 7B shows the total link strength. Bernstein CN (4014) and Ford AC (3975) are the main core authors in the co-citation network. Their works are frequently cited together, making them key nodes that connect the research context. Authors such as Mikocka-Walus A (1952), Sandborn WJ (1696), and Keefer L (1685) also demonstrate high link strength. They are important secondary core authors within the network. The co-citation network visualization results (Figure 7C) show that the reference authors in the field have formed a highly interconnected network, radiating multiple academic clusters: an IBD research cluster centered on Bernstein CN, a systematic review and meta-analysis cluster centered on Ford AC, and a psychological intervention and quality of life research cluster represented by Mikocka-Walus A and Graff LA. There is deep cross-disciplinary association among the clusters.

### 3.7 Analysis of co-cited reference journals

**Figure 8:**
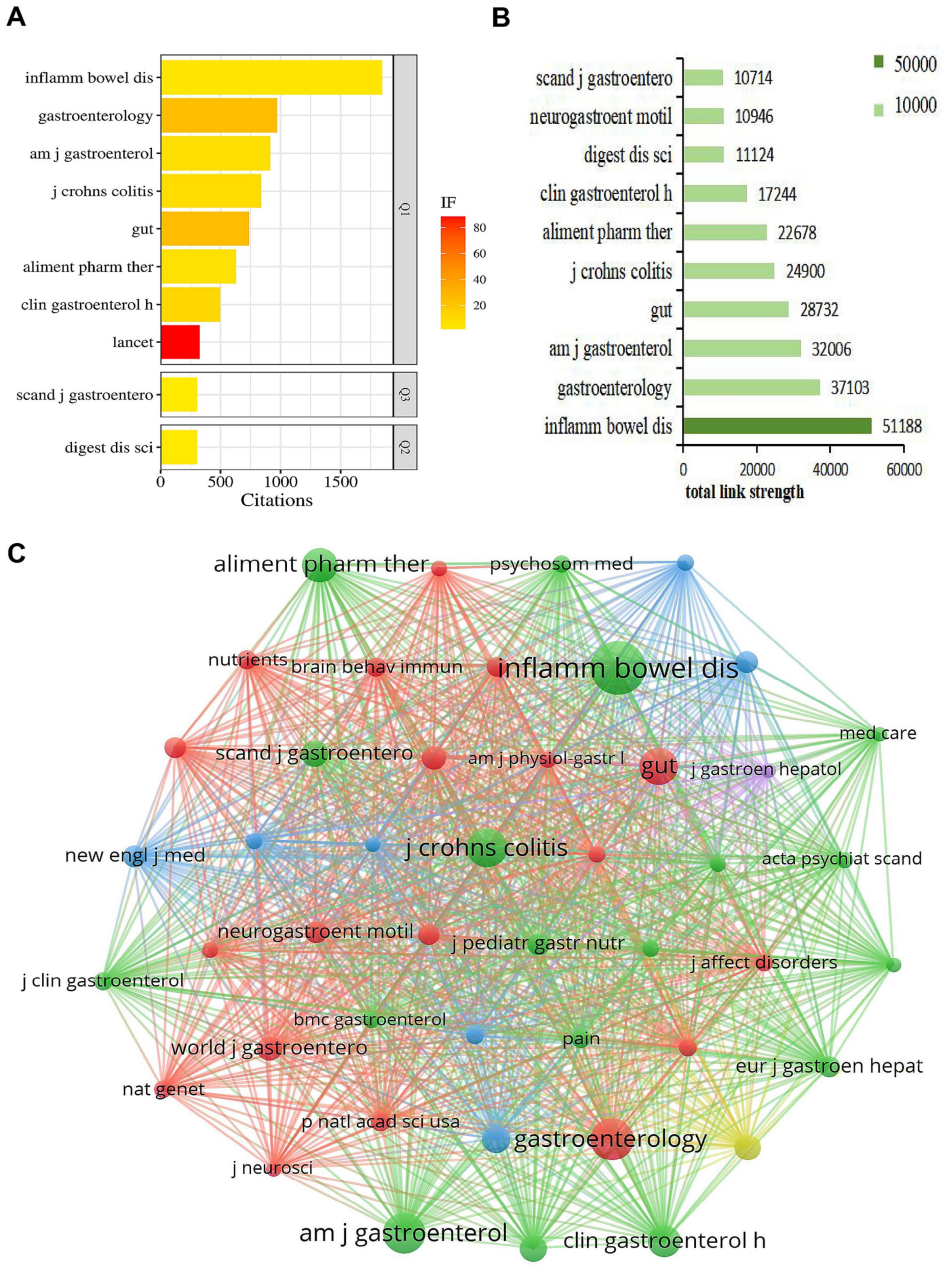
Analysis of co-cited reference journals. **(A)** Bar chart of the top 10 reference journals by total citation frequency. Bar color corresponds to the journal’s impact factor (IF), and bar length represents the journal’s total citation frequency. **(B)** Bar chart of the top 10 reference journals by total link strength. Bar color corresponds to the link strength range, and bar length represents the journal’s total link strength in the co-citation network. **(C)** Reference journal co-citation network map. Node size represents the journal’s citation frequency, node color represents different clusters, and connecting lines represent co-citation relationships between journals, with line thickness representing co-citation intensity.

The co-citation analysis of reference journals further traces the intellectual origin and academic dissemination context of UC comorbid with depression research, complementing the patterns observed in current publications. *Inflammatory Bowel Diseases* ranks first among reference journals in total citation frequency (Figure 8A), which highly corresponds to its publication scale and citation performance in the retrieved literature. This confirms that the journal serves both as a major publishing outlet for current research and as a foundational source of the field’s knowledge base. Leading gastroenterology journals such as *Gastroenterology*, the *American Journal of Gastroenterology*, the *Journal of Crohn’s and Colitis*, and *Gut* are the core tier for knowledge dissemination, among which *The Lancet* holds the highest IF, providing the highest quality evidence support for academic research. The total link strength analysis (Figure 8B) further confirms the academic hub status of core journals. The co-citation network visualization results (Figure 8C) reveals a highly interconnected structure among reference journals. The IBD professional journal cluster serves as the core carrier for knowledge inheritance in the field. Meanwhile, the psychiatry cluster represented by *Psychosomatic Medicine* and *Acta Psychiatrica Scandinavica* provides high-quality background support for depression research.

### 3.8 Analysis of hot keywords

**Figure 9:**
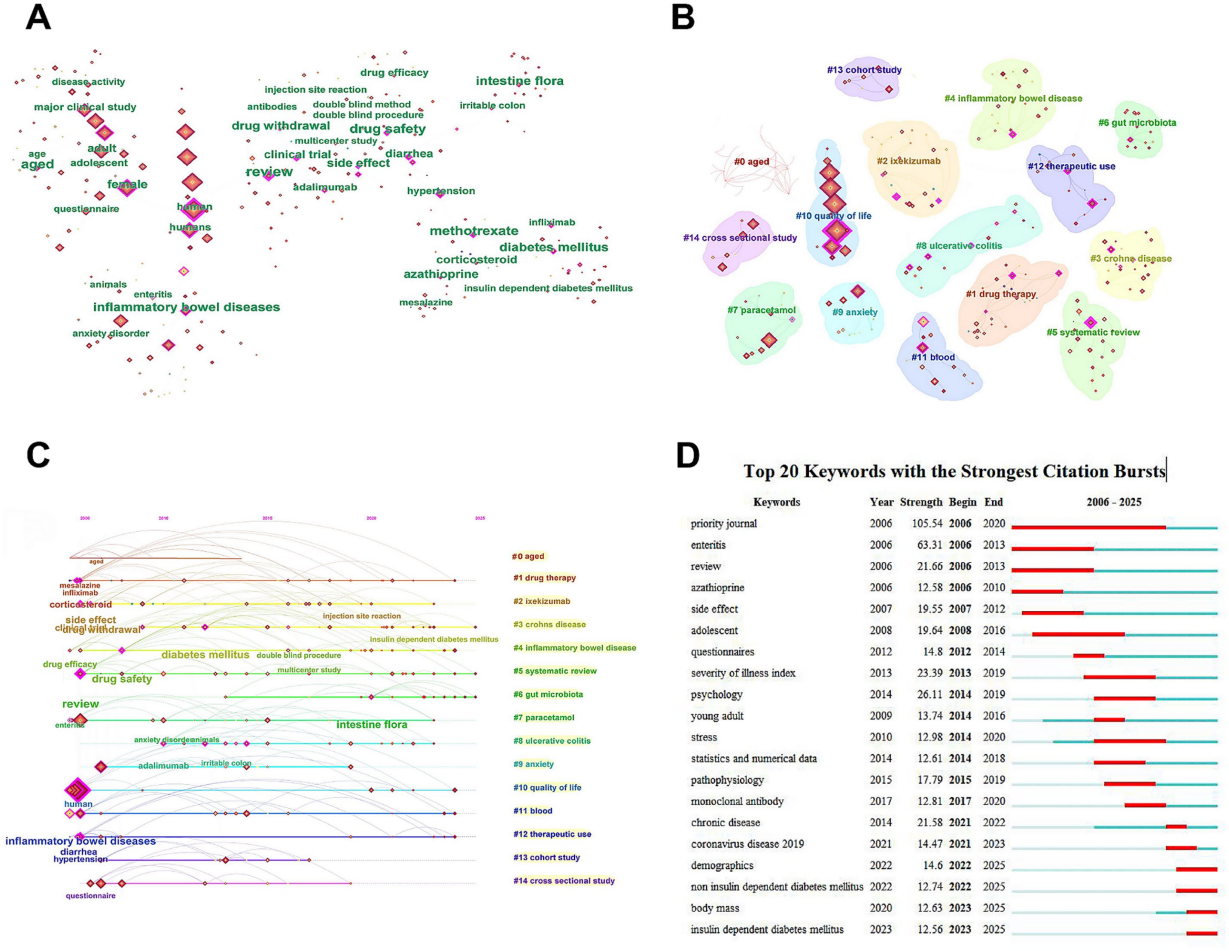
Analysis of hot keywords. **(A)** Keyword co-occurrence map. Node size represents the frequency of keyword occurrence, and node color represents betweenness centrality. **(B)** Keyword clustering map. Different colored areas represent different research theme clusters, connecting lines represent co-occurrence relationships between keywords, and cluster labels are automatically generated by the keyword co-occurrence network. **(C)** Keyword clustering timeline diagram. The horizontal axis represents the publication year of the literature, the vertical axis represents different research theme clusters, and node size corresponds to the frequency of keyword occurrence. **(D)** Keyword burst detection map. The red segment represents the time interval of keyword burst, the segment length represents the burst duration, and Strength represents the burst strength.

The keyword co-occurrence network analysis (Figure 9A) clearly shows the main topics and connection structure of the research. The network comprised 494 nodes and 521 edges (density = 0.0043), with a Modularity Q of 0.8589 and a weighted mean silhouette score of 0.9624, indicating robust cluster structures. In this network, inflammatory bowel disease, anxiety disorder, and depression are the biggest nodes, representing the main research subjects. Some keywords, such as drug safety, intestinal flora and methotrexate, are also obvious. Tables 4, 5 list the top 10 keywords based on occurrence frequency and betweenness centrality separately. The keyword clustering analysis (Figure 9B) finds several main groups, including #1 drug therapy, #2 ixekizumab, #3 crohn’s disease, #4 inflammatory bowel disease, #5 systematic review, #6 gut microbiota, #7 paracetamol, #8 ulcerative colitis, #9 anxiety, #10 quality of life, #11 blood, #12 therapeutic use, #13 cohort study and #14 cross sectional study. These clusters reflect the core research directions. The central focus is on IBD and neuropsychiatric comorbidities (clusters #3, #4, #8, and #9). Pharmacological interventions have been extensively investigated around this core (clusters #12 and #1), encompassing conventional symptomatic treatments (cluster #7), targeted biologic agents (cluster #2), and others. Clinical attention has been directed primarily toward patients’ quality of life (cluster #10) and blood-related markers (cluster #11). The methodological foundation underpinning these studies includes systematic reviews (cluster #5), cohort studies (cluster #13), and cross-sectional studies (cluster #14). Among the mechanistic explorations, gut microbiota (cluster #6) represents a key direction in the investigation of comorbidity mechanisms.

**Table 4:** Top 10 keywords by frequency.

Rank	Keywords	Citations
1	article	1,167
2	inflammatory bowel disease	1,147
3	humans	1,069
4	female	1,035
5	male	1,019
6	adult	998
7	crohn disease	945
8	major clinical study	767
9	quality of life	732
10	anxiety	670

**Table 5:** Top 10 keywords by centrality.

Rank	Keywords	Centrality
1	side effect	0.58
2	drug safety	0.57
3	female	0.49
4	diarrhea	0.48
5	hypertension	0.46
6	methotrexate	0.44
7	humans	0.42
8	inflammatory bowel diseases	0.4
9	priority journal	0.38
10	adalimumab	0.29

The keyword timeline diagram (Figure 9C) shows the temporal evolution of each research theme. From 2006 to 2015, research largely focused on population-based cross-sectional studies. Between 2015 to 2020, increasing attention was paid to drug safety, side effects and diabetes. Since 2020, intestinal flora and quality of life have remained consistently prominent research topics. The burst detection analysis (Figure 9D) identified the changes in research hotspots and frontier trends. Early burst keywords (2006–2013) were mainly enteritis and review, corresponding to the foundational stage of research. In the mid-term (2014–2020), keywords such as stress, psychology, and monoclonal antibody burst, reflecting the expansion of research toward psychological mechanisms and biologic therapy directions. Recently, keywords such as coronavirus disease, demographics, non-insulin dependent diabetes mellitus (NIDDM), and insulin dependent diabetes mellitus (IDDM) have high burst strength and long duration, representing frontier research directions in UC comorbid with depression. Qualitative analysis of the clinical trial records retrieved from the supplementary PubMed search (Supplementary Table S4) revealed that psychological/behavioral interventions (32.1%) constituted the predominant research category in clinical studies on the comorbidity. Observational studies also accounted for a considerable proportion (25.0%). In contrast, biological and pharmacological intervention studies were heterogeneously distributed across various directions, including probiotics, vitamin D, and 5-hydroxytryptophan (5-HTP).

## 4 Discussion

### 4.1 General information

There is a complex bidirectional influence mechanism between UC and depression. Genome-wide association studies have shown significant genetic locus overlap between key susceptibility genes for UC and depression (23). Hippocampal damage is an important central mechanism mediating inflammation-related depression (24). Recent studies have found that gut microbiota dysbiosis is a key biological link connecting UC and depression. UC patients have gut microbiota dysbiosis, characterized by reduced alpha diversity, decreased production of short-chain fatty acids (SCFA), and abnormal tryptophan metabolism (25, 26). Similarly, depressed patients exhibit similar gut microbiota changes, and studies have found that UC patients with comorbid depression have significantly more severe dysbiosis than patients with UC alone (27). HPA axis dysfunction is a core hub connecting depression and immune activation (28). Depressed patients often have HPA axis overactivation, i.e., “glucocorticoid resistance.” In UC patients, glucocorticoid resistance further weakens endogenous anti-inflammatory feedback, resulting in persistent amplification of intestinal inflammation (29).

Driven by multi-dimensional mechanistic research, academic output in the field of UC comorbid with depression has shown rapid growth. However, systematic quantitative assessments of the literature growth patterns and research frontiers are still lacking. Based on this, this study used the WOSCC and Scopus to conduct a systematic bibliometric and visualization analysis of research literature in the field of UC comorbid with depression from 2006 to 2025, supplemented by PubMed for additional qualitative insights. The results showed that the publication volume in this field has continued to grow, indicating that UC comorbid with depression has become a research hotspot in the intersection of gastroenterology and psychiatry. There were two significant increases in publication volume in 2013 and 2021. The growth around 2013 was mainly due to the gradual establishment of the gut-brain axis theory (30). This theory revealed that there is a direct pathological interaction between UC and depression, triggering in-depth research in the academic community. The rapid growth after 2021 may be closely related to the intestinal burden and increased psychological stress caused by the coronavirus disease 2019 (COVID-19) pandemic (31, 32).

For countries, the United States, the United Kingdom and Canada have the most papers. The United States has both high publication volume and citation frequency. It acts as a key country for research on UC combined with depression. The global research community now builds a cooperation network. European and American countries form the main part of this network. Countries like China, India and Australia also take part as important research members. Although China has published many papers, its academic influence is low, suggesting room for better design and deeper exploration. At the journal level, *Inflammatory Bowel Diseases* is the main journal for this research. *The Lancet Gastroenterology and Hepatology* and *Gut Microbes* published high-quality research results. The journal distribution in this field is centered on gastroenterology, and is deeply connected to psychiatry journals such as *Frontiers in Psychiatry* and *Journal of Psychosomatic Research*. This situation shows that this research field combines different medical subjects.

For authors, scholars from the United States and Canada have made significant contributions. The research teams led by Bernstein CN, Mikocka-Walus A, and Ford AC have the most papers and citations. Bernstein CN was an early researcher who paid close attention to the fact that mental problems like anxiety and depression are common in IBD patients (33), and to propose that the gut-brain axis plays a big part. Mikocka-Walus A specializes in neuropsychology. Her research has shown that psychological treatments, including acceptance and commitment therapy (ACT), confer benefits in UC patients (34),thereby offering key evidence for interdisciplinary disease management. The Ford AC team has focused on mining evidence-based medicine evidence for UC comorbid with depression, Their work explains the connection between depression and the start or worsening of UC (35).

### 4.2 Knowledge base

Reference co-citation analysis identifies the foundational theoretical basis for studies on UC combined with depression (36, 37). The highly cited literature is dominated by guidelines and systematic reviews, which integrate existing evidence and provide methodological frameworks for subsequent research. Cluster analysis reveals that most research centers on several key contents. These contents include “coping,” “pain management,” “perceived stress,” and “psychiatry.” Notably cluster #14 (suicide) indicates clinical attention to severe psychological outcomes in UC patients. Timeline analysis shows that early research primarily focused on abdominal pain and rectal/anal pain, which were recognized not merely as somatic symptoms but as manifestations arising from the interplay of inflammation, neural perception, and psychological state (38–40). During this stage, however, the impact of depression on UC had not yet attracted substantial research interest. With the penetration of psychiatric concepts, the impact of perceived stress from depression on UC activity received more attention. After 2020, the stress and suicide risk precipitated by the COVID-19 pandemic increased sharply, prompting a surge of investigations.

Among the cited authors, Bernstein CN, Mikocka-Walus A, and Ford AC set up the core knowledge system of the field through continuous and extensive original research. Scholars such as Mikocka-Walus A and Graff LA have focused on psychological intervention and quality of life issues, effectively filling the research gaps in clinical application. For the cited journals, *Inflammatory Bowel Diseases* is the core carrier of knowledge accumulation and academic dissemination in the field. Journals such as *The Lancet Gastroenterology and Hepatology*, *Gut Microbes*, *Gut*, and *Journal of Crohn’s and Colitis continuously* produce high-quality evidence. Psychiatry journals such as *Psychosomatic Medicine* and *Acta Psychiatrica Scandinavica* also play a key role in the co-citation network. They give mental health theory support for research on UC and depression comorbidity research.

### 4.3 Hotspots and frontiers

Keyword analysis shows the focus main topics evolution patterns and frontier directions of a research field (41). Epidemiological research is one of the prominent hot topic in this field. In recent years multiple cross-sectional studies have confirmed that depression significantly increases the risk of UC and this risk association can persist for several years following the diagnosis of depression (42–44). These findings changed the unidirectional perspective and confirmed a significant bidirectional relationship between UC and depression. High-frequency keywords such as “female,” “male,” “adult,” and “adolescent” highlight the primary study populations of interest in comorbidity research. Existing studies have reported a higher prevalence of depression in female UC patients compared to their male counterparts (45) while the increasing incidence of UC in adolescents has been linked to an elevated risk of depression in this population (46, 47). Given that depressive disorders in some adult UC patients may originate during adolescence (48) the establishment of long-term psychological monitoring mechanisms for adolescent patients warrants further investigation. The emergence of high-frequency keywords such as “major clinical study” and “quality of life” indicates a progressive shift in research focus toward patient-centered clinical investigations aimed at improving quality of life (49). Keywords including “side effect” and “drug safety” suggest scholarly attention to the risks associated with polypharmacy in comorbid conditions. “Diarrhea” not only represents a core symptom of UC but also constitutes a commonly reported adverse event associated with targeted therapies while “hypertension” may signal metabolic abnormalities arising from long-term chronic disease interventions (50). Collectively these terms serve as important indicators for drug safety monitoring.

Burst detection analysis identified “coronavirus disease,” “NIDDM,” and “IDDM” as the keywords with the highest burst strength in the most recent period, representing the frontier research directions in this field. The outbreak of COVID-19 has led to an increased prevalence of comorbid depression in patients with UC. SARS-CoV-2 can directly invade the gastrointestinal system and increase the risk of UC activity, while pandemic-related isolation and psychological stress may exacerbate depression (51). The bursts of diabetes-related terms reflect growing research interest in the interconnections among UC, depression, and metabolic disorders. Studies have shown that UC patients with comorbid depression are at significantly increased risk of developing insulin-dependent diabetes mellitus (52, 53). UC and depression may jointly contribute to diabetes risk through shared genetic susceptibility loci, HPA axis overactivation, and persistent chronic inflammation (54, 55). The burst patterns of the aforementioned keywords suggest that research in this field is expanding from the traditional gut-brain axis toward a metabolic framework.

The supplementary search in PubMed reveals that clinical research on UC-depression comorbidity has developed rapidly over the past two decades. Around 2010, a few intervention attempts for the comorbidity emerged in this field (56). From 2014, a substantial number of randomized controlled trials(RCT) of psychological therapies were published, with studies reporting that cognitive behavioral therapy (CBT), ACT, and mindfulness-based cognitive therapy could alleviate symptoms and improve quality of life in IBD patients to varying degrees (34, 57, 58). After 2018, with a deeper understanding of gut–brain axis mechanisms, intervention strategies expanded to encompass biological approaches, including nutrition, microbiome modulation, and pharmacotherapy. During this period, promising efficacy was reported for agents such as vitamin D, probiotics, and 5-hydroxytryptophan (5-HTP) (59–61). Of note, dynamic observations of the interplay among disease activity, inflammatory markers, and psychological factors have been a consistent thread throughout the research timeline (62).

### 4.4 Advantages and limitations

This study used bibliometric and visualization analysis to systematically sort out the overall research pattern in the field of UC comorbid with depression. Compared with previous reviews that focused only on a single theme, this study presented the overall development context of the field from multiple dimensions, including publication trends, country cooperation, author contribution, reference co-citation, and keyword co-occurrence, from epidemiological description to clinical intervention. This study included the latest research literature and used keyword burst detection to accurately identify the latest hotspots and frontier directions, providing a more timely roadmap and topic selection guidance for subsequent research. Compared with previous similar studies, this study simultaneously incorporated both the WOSCC and Scopus databases. WOSCC excels in its coverage of classic core journals and depth of citation data, while Scopus demonstrates strengths in journal breadth and the inclusion of emerging literature. The combination of the two databases enabled complementary data sources, contributing to more comprehensive and robust findings.

Several limitations of this study should be acknowledged. This study used the WOSCC and Scopus as the primary databases for literature retrieval, supplemented by PubMed for validation. Although these databases cover high-impact English-language literature in this field, Embase was not included due to limitations in metadata completeness, which may have resulted in the omission of some relevant studies. In addition, as this study set a specific retrieval time window to reflect research trends, recently published articles may have received limited citations, potentially leading to the underestimation of certain emerging topics. The study primarily employed CiteSpace and VOSviewer for visualization and co-citation analysis. However, these tools do not allow for quantitative or qualitative appraisal of study design, making it difficult to differentiate methodological quality across studies (63, 64). Future research could incorporate specialized databases such as Embase to enhance the coverage of pharmacological and mechanistic studies.

## 5 Conclusion

In this study, we employed bibliometric methods to systematically delineate the research history, thematic hotspots, and frontier trends in the field of ulcerative colitis comorbid with depression. As a significant interdisciplinary domain bridging gastroenterology and psychiatry, this field has witnessed sustained growth in publication output over the past two decades. External environmental stressors and intrinsic metabolic dysregulation are jointly reshaping the frontier landscape of the field. Beyond traditional epidemiological investigations, patient quality of life and drug safety have emerged as current research priorities. The exploration of diverse intervention strategies—including psychological therapies, nutritional support, microbiome modulation, and biomarker-guided targeted therapies—has expanded the therapeutic armamentarium for clinical management. However, the evidence base for these interventions remains limited in terms of the number of studies available. Future research should focus on generating more robust clinical evidence and promoting multidisciplinary collaborative care, with the goal of improving patients’ quality of life and long-term prognosis.

## Data Availability

Publicly available datasets were analyzed in this study. The raw bibliometric data were retrieved from the publicly available Web of Science Core Collection and Scopus databases. The datasets generated during this study are not deposited in a public repository but are available from the corresponding author upon reasonable request.

## References

[1] Eggermont E Gecse K Krugliak Cleveland N Petersen F Sabino J D'Hoore A . Ulcerative colitis: moving beyond the mucosal dogma. Lancet Gastroenterol Hepatol. (2026) 11:163–74. doi: 10.1016/S2468-1253(25)00263-8, 41173014

[2] Catalano M Mini E Nobili S Vascotto IA Ravizza D Amorosi A . Ulcerative colitis and colorectal cancer: pathogenic insights and precision strategies for prevention and treatment. World J Gastrointest Oncol. (2025) 17:108514. doi: 10.4251/wjgo.v17.i10.108514, 41114102 PMC12531786

[3] Zhang L Zhang X Su T Xiao T Xu H Zhao S. Colorectal cancer risk in ulcerative colitis: an updated population-based systematic review and meta-analysis. EClinicalMedicine. (2025) 84:103269. doi: 10.1016/j.eclinm.2025.103269, 40496880 PMC12150054

[4] Hracs L Windsor JW Gorospe J Global IBD Visualization of Epidemiology Studies in the 21st Century (GIVES-21) Research Group. Global evolution of inflammatory bowel disease across epidemiologic stages. Nature. (2025) 642:458–66. doi: 10.1038/s41586-025-08940-0 40307548 PMC12158780

[5] Le Berre C Honap S Peyrin-Biroulet L. Ulcerative colitis. Lancet. (2023) 402:571–84. doi: 10.1016/S0140-6736(23)00966-2, 37573077

[6] Randlová K Marešová P Režný L Hruška J Srdanović S Guðmundsson GH . Cost-effectiveness of treatment and care of patients with gastrointestinal inflammatory diseases: a systematic review. J Med Econ. (2026) 29:919–39. doi: 10.1080/13696998.2026.2637360, 41861399

[7] Massironi S Pigoni A Vegni EAM Keefer L Dubinsky MC Brambilla P . The burden of psychiatric manifestations in inflammatory bowel diseases: a systematic review with Meta-analysis. Inflamm Bowel Dis. (2025) 31:1441–59. doi: 10.1093/ibd/izae206, 39270637

[8] Luo K Zhang M Tu Q Li J Wang Y Wan S . From gut inflammation to psychiatric comorbidity: mechanisms and therapies for anxiety and depression in inflammatory bowel disease. J Neuroinflammation. (2025) 22:149. doi: 10.1186/s12974-025-03476-6, 40462068 PMC12135330

[9] Barberio B Zamani M Black CJ Savarino EV Ford AC. Prevalence of symptoms of anxiety and depression in patients with inflammatory bowel disease: a systematic review and meta-analysis. Lancet Gastroenterol Hepatol. (2021) 6:359–70. doi: 10.1016/S2468-1253(21)00014-5, 33721557

[10] Hinnant L Rios Villacorta N Chen E Bacchus D Dotson J Greywoode R . Consensus statement on managing anxiety and depression in individuals with inflammatory bowel disease. Inflamm Bowel Dis. (2025) 31:1248–55. doi: 10.1093/ibd/izae151, 39173019 PMC12069991

[11] Kochar B Barnes EL Long MD Cushing KC Galanko J Martin CF . Depression is associated with more aggressive inflammatory bowel disease. Am J Gastroenterol. (2018) 113:80–5. doi: 10.1038/ajg.2017.423, 29134965 PMC5962285

[12] Ji Y Li H Dai G Zhang X Ju W. Systematic review and meta-analysis: impact of depression on prognosis in inflammatory bowel disease. J Gastroenterol Hepatol. (2024) 39:1476–88. doi: 10.1111/jgh.16568, 38655853

[13] Qian Y Chen Y Liu L Wu T Chen X Ma G. Depression and anxiety in inflammatory bowel disease: mechanisms and emerging therapeutics targeting the microbiota-gut-brain axis. Front Immunol. (2025) 16:1676160. doi: 10.3389/fimmu.2025.1676160, 41280932 PMC12634526

[14] Yuan X Chen B Duan Z Xia Z Ding Y Chen T . Depression and anxiety in patients with active ulcerative colitis: crosstalk of gut microbiota, metabolomics and proteomics. Gut Microbes. (2021) 13:1987779. doi: 10.1080/19490976.2021.1987779, 34806521 PMC8632339

[15] Agirman G Yu KB Hsiao EY. Signaling inflammation across the gut-brain axis. Science. (2021) 374:1087–92. doi: 10.1126/science.abi6087, 34822299

[16] van Eck NJ Waltman L. Software survey: VOSviewer, a computer program for bibliometric mapping. Scientometrics. (2010) 84:523–38. doi: 10.1007/s11192-009-0146-3, 20585380 PMC2883932

[17] Chen C. CiteSpace II: detecting and visualizing emerging trends and transient patterns in scientific literature. J Am Soc Inf Sci Technol. (2006) 57:359–77. doi: 10.1002/asi.20317

[18] Chen G Yan Z Wang Y Tao Q. Rheumatoid arthritis and fibromyalgia syndrome: a bibliometric and bioinformatics perspective on comorbidity research. J Multidiscip Healthc. (2025) 18:6811–27. doi: 10.2147/JMDH.S547111, 41142638 PMC12553343

[19] Hassan-Montero Y De-Moya-Anegón F Guerrero-Bote VP. SCImago Graphica: a new tool for exploring and visually communicating data. El Prof Inf. (2022) 31:e310502. doi: 10.3145/epi.2022.sep.02

[20] Barberio B Zamani M Black CJ Savarino EV Ford AC. Prevalence of symptoms of anxiety and depression in patients with inflammatory bowel disease: a systematic review and meta-analysis. Lancet Gastroenterol Hepatol. (2021) 6:359–70. doi: 10.1016/S2468-1253(21)00014-5, 33721557

[21] Bisgaard TH Allin KH Keefer L Ananthakrishnan AN Jess T. Depression and anxiety in inflammatory bowel disease: epidemiology, mechanisms and treatment. Nat Rev Gastroenterol Hepatol. (2022) 19:717–26. doi: 10.1038/s41575-022-00634-6, 35732730

[22] Mikocka-Walus A Knowles SR Keefer L Graff L. Controversies revisited: a systematic review of the comorbidity of depression and anxiety with inflammatory bowel diseases. Inflamm Bowel Dis. (2016) 22:752–62. doi: 10.1097/MIB.0000000000000620, 26841224

[23] Tripathi U Stern Y Dagan I Nayak R Romanovsky E Zittan E . From genome-wide SNPs to neuroimmune crosstalk: mapping the genetic landscape of IBD and its brain overlap. Biology. (2025) 14:1433. doi: 10.3390/biology14101433 41154835 PMC12562180

[24] Zonis S Pechnick RN Ljubimov VA Mahgerefteh M Wawrowsky K Michelsen KS . Chronic intestinal inflammation alters hippocampal neurogenesis. J Neuroinflammation. (2015) 12:65. doi: 10.1186/s12974-015-0281-0, 25889852 PMC4403851

[25] Newman KL Standke AK James G Vendrov KC Inohara N Bergin IL . Miniature bioreactor arrays for modeling functional and structural dysbiosis in inflammatory bowel disease. Gut Microbes. (2026) 18:2604875. doi: 10.1080/19490976.2025.2604875, 41427586 PMC12810045

[26] Houshyar Y Zhang F Tavakoli P Grimm MC Hold GL. Neglected kingdoms: the gut virome, mycobiome and their role in inflammatory bowel disease. Gut Microbes. (2026) 18:2653288. doi: 10.1080/19490976.2026.2653288, 41928387 PMC13051595

[27] Lee J Oh SJ Ha E Shin GY Kim HJ Kim K . Gut microbial and human genetic signatures of inflammatory bowel disease increase risk of comorbid mental disorders. NPJ Genom Med. (2024) 9:52. doi: 10.1038/s41525-024-00440-w, 39472439 PMC11522461

[28] Feng X Jia M Cai M Zhu T Hashimoto K Yang JJ. Central-peripheral neuroimmune dynamics in psychological stress and depression: insights from current research. Mol Psychiatry. (2025) 30:4881–98. doi: 10.1038/s41380-025-03085-y, 40610703 PMC12436188

[29] Tchinda Defo SH Moussa D Bouvourné P Guédang Nyayi SD Woumitna GC Kodji K . Unpredictable chronic mild stress induced anxio-depressive disorders and enterobacteria dysbiosis: potential protective effects of Detarium microcarpum. J Ethnopharmacol. (2025) 337:118940. doi: 10.1016/j.jep.2024.118940, 39423942

[30] Bonaz BL Bernstein CN. Brain-gut interactions in inflammatory bowel disease. Gastroenterology. (2013) 144:36–49. doi: 10.1053/j.gastro.2012.10.003, 23063970

[31] Cheema M Mitrev N Hall L Tiongson M Ahlenstiel G Kariyawasam V. Depression, anxiety and stress among patients with inflammatory bowel disease during the COVID-19 pandemic: Australian national survey. BMJ Open Gastroenterol. (2021) 8:e000581. doi: 10.1136/bmjgast-2020-000581, 33579729 PMC7883604

[32] Mosli M Alourfi M Alamoudi A Hashim A Saadah O Al Sulais E . A cross-sectional survey on the psychological impact of the COVID-19 pandemic on inflammatory bowel disease patients in Saudi Arabia. Saudi J Gastroenterol. (2020) 26:263–71. doi: 10.4103/sjg.SJG_220_20, 32567580 PMC7739990

[33] Walker JR Ediger JP Graff LA Greenfeld JM Clara I Lix L . The Manitoba IBD cohort study: a population-based study of the prevalence of lifetime and 12-month anxiety and mood disorders. Am J Gastroenterol. (2008) 103:1989–97. doi: 10.1111/j.1572-0241.2008.01980.x, 18796096

[34] Naude C Skvarc D Maunick B Evans S Romano D Chesterman S . Acceptance and commitment therapy for adults living with inflammatory bowel disease and distress: a randomized controlled trial. Am J Gastroenterol. (2024) 120:1839–51. doi: 10.14309/ajg.0000000000003032, 39162706 PMC12282595

[35] Fairbrass KM Lovatt J Barberio B Yuan Y Gracie DJ Ford AC. Bidirectional brain-gut axis effects influence mood and prognosis in IBD: a systematic review and meta-analysis. Gut. (2022) 71:1773–80. doi: 10.1136/gutjnl-2021-325985, 34725197

[36] Wang Y Wang Z Lou P Ni H Yang Y Chen G . Research trends and hotspots in JAK inhibitors for ulcerative colitis: a bibliometric analysis from 2015 to 2024. J Multidiscip Healthc. (2025) 18:6755–71. doi: 10.2147/JMDH.S548268, 41127384 PMC12539411

[37] Shi H Yang K Jiang W You Y Men X Wang L . Collision of hydrogels, chronic wounds, and nanotechnology: a bibliometric analysis from 2009 to 2024. Colloids Surf B Biointerfaces. (2026) 262:115549. doi: 10.1016/j.colsurfb.2026.115549, 41702150

[38] Tan WW Liu ZX Liu XY Zhang WB Zheng L Zhang YL . Abdominal pain in inflammatory bowel disease-epidemiology, pathophysiology, and management: a narrative review. Pain Ther. (2024) 13:1447–69. doi: 10.1007/s40122-024-00672-9, 39466554 PMC11543983

[39] Makharia GK. Understanding and treating abdominal pain and spasms in organic gastrointestinal diseases: inflammatory bowel disease and biliary diseases. J Clin Gastroenterol. (2011) 45:S89–93. doi: 10.1097/MCG.0b013e31821fbd82, 21666426

[40] Grundy D Al-Chaer ED Aziz Q Collins SM Ke M Taché Y . Fundamentals of neurogastroenterology: basic science. Gastroenterology. (2006) 130:1391–411. doi: 10.1053/j.gastro.2005.11.060, 16678554

[41] Xi Z Zhu L Peng X. Hot topics and trends in acupuncture for herpes zoster from 2015 to 2025: a bibliometric and knowledge graph analysis. Front Med. (2026) 13:1748878. doi: 10.3389/fmed.2026.1748878, 41889492 PMC13012937

[42] Mikocka-Walus A Pittet V Rossel JB von Känel R Swiss IBD Cohort Study Group. Symptoms of depression and anxiety are independently associated with clinical recurrence of inflammatory bowel disease. Clin Gastroenterol Hepatol. (2016) 14:829–835.e1. doi: 10.1016/j.cgh.2015.12.045 26820402

[43] Frolkis AD Vallerand IA Shaheen AA Lowerison MW Swain MG Barnabe C . Depression increases the risk of inflammatory bowel disease, which may be mitigated by the use of antidepressants in the treatment of depression. Gut. (2019) 68:1606–12. doi: 10.1136/gutjnl-2018-317182, 30337374

[44] Piovani D Armuzzi A Bonovas S. Association of Depression with Incident Inflammatory Bowel Diseases: a systematic review and Meta-analysis. Inflamm Bowel Dis. (2024) 30:573–84. doi: 10.1093/ibd/izad109, 37300511 PMC10988103

[45] Sempere L Bernabeu P Cameo J Gutierrez A Laveda R Garcia MF . Evolution of the emotional impact in patients with early inflammatory bowel disease during and after COVID-19 lockdown. Gastroenterol Hepatol. (2022) 45:123–33. doi: 10.1016/j.gastrohep.2021.03.004, 34023470 PMC8807180

[46] Vodnar DC Mitrea L Teleky BE Szabo K Călinoiu LF Nemeş SA . Coronavirus disease (COVID-19) caused by (SARS-CoV-2) infections: a real challenge for human gut microbiota. Front Cell Infect Microbiol. (2020) 10:575559. doi: 10.3389/fcimb.2020.575559, 33363049 PMC7756003

[47] Kuenzig ME Fung SG Marderfeld L Mak JWY Kaplan GG Ng SC . Twenty-first century trends in the global epidemiology of pediatric-onset inflammatory bowel disease: systematic review. Gastroenterology. (2022) 162:1147–1159.e4. doi: 10.1053/j.gastro.2021.12.282, 34995526

[48] Thavamani A Umapathi KK Khatana J Gulati R. Burden of psychiatric disorders among pediatric and Young adults with inflammatory bowel disease: a population-based analysis. Pediatr Gastroenterol Hepatol Nutr. (2019) 22:527–35. doi: 10.5223/pghn.2019.22.6.527, 31777718 PMC6856511

[49] Calviño-Suárez C Ferreiro-Iglesias R Bastón-Rey I Barreiro-de Acosta M. Role of quality of life as endpoint for inflammatory bowel disease treatment. Int J Environ Res Public Health. (2021) 18:7159. doi: 10.3390/ijerph18137159, 34281095 PMC8296948

[50] Solitano V Vuyyuru SK MacDonald JK Zayadi A Parker CE Narula N . Efficacy and safety of advanced oral small molecules for inflammatory bowel disease: systematic review and meta-analysis. J Crohns Colitis. (2023) 17:1800–16. doi: 10.1093/ecco-jcc/jjad100, 37317532

[51] Shaffer SR. How did COVID-19 affect mental health and access to Care in Persons with Inflammatory Bowel Disease. J Can Assoc Gastroenterol. (2023) 7:115–20. doi: 10.1093/jcag/gwad033, 38314179 PMC10836987

[52] Samuelsson J Bertilsson R Bülow E Carlsson S Åkesson S Eliasson B . Autoimmune comorbidity in type 1 diabetes and its association with metabolic control and mortality risk in young people: a population-based study. Diabetologia. (2024) 67:679–89. doi: 10.1007/s00125-024-06086-8, 38252314 PMC10904419

[53] Fousekis F Lamprou A Saridi M Mastorogianni IN Mpakogiannis K Lianos GD . Metabolic disorders and inflammatory bowel diseases: unraveling shared pathways and clinical interactions. Meta. (2026) 16:181. doi: 10.3390/metabo16030181, 41893332 PMC13028057

[54] Wang K Baldassano R Zhang H Qu HQ Imielinski M Kugathasan S . Comparative genetic analysis of inflammatory bowel disease and type 1 diabetes implicates multiple loci with opposite effects. Hum Mol Genet. (2010) 19:2059–67. doi: 10.1093/hmg/ddq078, 20176734 PMC2860894

[55] Slyepchenko A Maes M Jacka FN Köhler CA Barichello T McIntyre RS . Gut microbiota, bacterial translocation, and interactions with diet: pathophysiological links between major depressive disorder and non-communicable medical comorbidities. Psychother Psychosom. (2017) 86:31–46. doi: 10.1159/000448957 27884012

[56] Elkjaer M Shuhaibar M Burisch J Bailey Y Scherfig H Laugesen B . E-health empowers patients with ulcerative colitis: a randomised controlled trial of the web-guided 'constant-care' approach. Gut. (2010) 59:1652–61. doi: 10.1136/gut.2010.220160, 21071584

[57] Szigethy E Bujoreanu SI Youk AO Weisz J Benhayon D Fairclough D . Randomized efficacy trial of two psychotherapies for depression in youth with inflammatory bowel disease. J Am Acad Child Adolesc Psychiatry. (2014) 53:726–35. doi: 10.1016/j.jaac.2014.04.014, 24954822 PMC4104185

[58] Hu S Xu D Zhu C Wang Y Li S Chen Y . Impact of anxiety, depression and online mindfulness on IBD patients' quality of life: a web-based cross-sectional survey and randomized pilot study. BMC Psychiatry. (2025) 25:1063. doi: 10.1186/s12888-025-07426-7, 41199235 PMC12593856

[59] Sharifi A Vahedi H Nedjat S Mohamadkhani A Hosseinzadeh Attar MJ. Vitamin D decreases Beck depression inventory score in patients with mild to moderate ulcerative colitis: a double-blind randomized placebo-controlled trial. J Diet Suppl. (2019) 16:541–9. doi: 10.1080/19390211.2018.1472168, 29958055

[60] Liu X Zhou H Zhang J Li R Liang J. Brain-gut co-management: probiotic LAB improves mental health and further reduces disease activity in ulcerative colitis patients with emotional disturbance. Nutr Neurosci. (2025) 28:1511–22. doi: 10.1080/1028415X.2025.2527224, 40629893

[61] Truyens M Lobatón T Ferrante M Bossuyt P Vermeire S Pouillon L . Effect of 5-Hydroxytryptophan on fatigue in quiescent inflammatory bowel disease: a randomized controlled trial. Gastroenterology. (2022) 163:1294–1305.e3. doi: 10.1053/j.gastro.2022.07.052, 35940251

[62] Hu S Xu D Zhu C Wang Y Li S Chen Y . Impact of anxiety, depression and online mindfulness on IBD patients' quality of life: a web-based cross-sectional survey and randomized pilot study. BMC Psychiatry. (2025) 25:1063. doi: 10.1186/s12888-025-07426-7, 41199235 PMC12593856

[63] Zhao Y Chen GY Fang M. Research trends of rheumatoid arthritis and depression from 2019 to 2023: a bibliometric analysis. J Multidiscip Healthc. (2024) 17:4465–74. doi: 10.2147/JMDH.S478748, 39308796 PMC11416121

[64] Wang YF Ma LJ Han YX Chen GY Ma X. Global trends and hotspots of artificial intelligence in pain management: a bibliometric analysis. Digit Health. (2026) 12:20552076261460708. doi: 10.1177/20552076261460708, 42311841 PMC13269973

