# Supplementary material for: Research hotspots, context, and future directions of ulcerative colitis comorbid with depression from 2006 to 2025: a bibliometric and visualization analysis
Source: Front Med (Lausanne). 2026 Aug 5;13:1868648. doi: 10.3389/fmed.2026.1868648 (PMC13485740; doi:10.3389/fmed.2026.1868648)
Supplement: Supplementary file 1 [file Data_Sheet_1.PDF]

Supplementary Material

Supplementary Figures and Tables

Table S1. Detailed search strategies and inclusion criteria across databases.

Database	WOSCC	Scopus	PubMed
Search Date	2026/4/2	2026/4/2	2026/6/26
Search Query	TS=("ulcerative colitis" AND (depression OR "depressive disorder" OR "depressive symptom*"))	TITLE-ABS-KEY("ulcerative colitis" AND (depression OR "depressive disorder" OR "depressive symptom*"))	("ulcerative colitis"[tiab] AND (depression[tiab] OR "depressive disorder"[tiab] OR "depressive symptom*"[tiab]))
Type	Article, Review Article	Article, Review Article	Clinical Trial
Time	2006.1.1-2025.12.31	2006.1.1-2025.12.31	2006.1.1-2025.12.31
Language	English	English	English
Index	SCI-E, SSCI	Scopus	MEDLINE
Export Settings	Plain Text File<br>Tab-delimited File	CSV	text
Results	1028	1595	28

Table S2. Annual Publication Distribution and Average Annual Citations of Retrieved Literature in WOSCC

Year	citation counts	publications	Average annual citations
2006	10	20060	1003.00
2007	14	30105	1584.47
2008	16	32128	1784.89
2009	16	34153	2009.00
2010	17	34170	2135.63
2011	13	28154	1876.93
2012	15	30180	2155.71
2013	28	56364	4335.69
2014	31	64448	5370.67
2015	39	86645	7876.82
2016	33	68544	6854.40
2017	65	131105	14567.22
2018	61	125116	15639.50
2019	63	129216	18459.43
2020	88	177760	29626.67
2021	88	177848	35569.60
2022	115	232530	58132.50
2023	106	165886	55295.33
2024	93	188232	94116.00
2025	118	240975	240975

Table S3. Annual Publication Distribution and Average Annual Citations of Retrieved Literature in Scopus

Year	citation counts	publications	Average annual citations
2006	1347	12	67.35
2007	1046	15	55.05
2008	2343	21	130.17
2009	1813	19	106.65
2010	1833	22	114.56
2011	1591	29	106.07
2012	3110	32	222.14
2013	4320	55	332.31
2014	3390	51	282.50
2015	4546	62	413.27
2016	4025	50	402.50
2017	4570	76	507.78
2018	4133	70	516.63
2019	4060	87	580.00
2020	3511	95	585.17
2021	6364	169	1272.80
2022	4406	166	1101.50
2023	2766	177	922.00
2024	1448	195	724.00
2025	472	192	472

Average annual citations: Total citations of publications published in a given year divided by the number of natural years elapsed since publication.

Table S4. Summary of supplementary clinical trials identified from PubMed

Therapeutic Category	Count	Primary Study Designs	Core Intervention / Focus
Psychological / Behavioral Interventions	9	RCT (n=7), observational (n=1), clinical trial (n=1)	CBT, ACT, mindfulness, hypnotherapy, group psychotherapy
Observational Studies on Comorbidity	7	Observational (n=5), cross-sectional (n=1), prospective cohort (n=1)	Depression/anxiety and IBD disease activity, healthcare utilization, fatigue, sleep, relapse risk
Microbiome / Probiotics	3	RCT	Nano-curcumin, probiotic, four-strain probiotic
Nutritional Supplements	4	RCT	Vitamin D, spirulina, melatonin
Pharmacological / Biological Therapy	3	RCT (n=1), observational (n=2)	5-HTP, tianeptine, mesalazine combination therapy
Traditional / Alternative Medicine	1	RCT	Herb-partitioned moxibustion
Digital Health	1	RCT	E-health "Constant-care" approach
